# Hollow Silica Particles: Recent Progress and Future Perspectives

**DOI:** 10.3390/nano10081599

**Published:** 2020-08-14

**Authors:** Jaswinder Sharma, Georgios Polizos

**Affiliations:** Roll-to-Roll Manufacturing Group, Energy and Transportation Science Division, Oak Ridge National Laboratory, Oak Ridge, TN 37831, USA

**Keywords:** hollow, mesoporous, silica, particles, synthesis, characterization, applications

## Abstract

Hollow silica particles (or mesoporous hollow silica particles) are sought after for applications across several fields, including drug delivery, battery anodes, catalysis, thermal insulation, and functional coatings. Significant progress has been made in hollow silica particle synthesis and several new methods are being explored to use these particles in real-world applications. This review article presents a brief and critical discussion of synthesis strategies, characterization techniques, and current and possible future applications of these particles.

## 1. Introduction

In recent years, hollow materials have attracted significant attention from scientists working in different fields. Hollow materials with different shapes, including rods, fibers, and spherical particles, have been synthesized [1,2,3,4,5]. Of these shapes, hollow particles (spherical in shape) are the most interesting class, with applications ranging from uses in energy storage to coatings. Different materials have been used to create hollow particles, such as ZnO, Si, TiO_2_, C, SiO_2_, and SnO_2_ [6,7,8,9,10,11,12,13,14]. Among these hollow particle options, hollow silica particles (HSPs) are highly sought after because of the low cost of the material and its synthesis process, their well-known chemistry, and their wide applications, ranging from energy storage to functional coatings [15,16,17,18,19,20,21,22,23]. A search for the keywords “hollow silica particles” on Web of Science returned 122 publications. It must be noted that the inherent nature of HSPs synthesis results in micro- or mesoporous shells. In this review, the use of word HSPs implies in most cases HSPs with a micro or mesoporous shell (silica shells having micro-or mesopores). Porosity of particles refers to the pores in the shell and not to the size of central hollow cavity of the HSPs. In the last three decades, several synthesis strategies have emerged, and recently several new applications of HSPs have been developed. Some review articles cover the synthesis and applications of HSPs [24,25,26], but considering the rapidly growing field and the new applications (e.g., thermal insulation and energy storage), an updated review article involving these new applications is required. In this minireview, the synthesis, characterization, and old (drug delivery, catalysis, functional coatings) and new (e.g., thermal insulation and energy storage) applications of HSPs are discussed, with critical commentary included. Future prospects and possible new applications of the HSPs are also discussed. Since the material properties depend on the size at the nano-microscale, the scope of this review is confined to the HSPs below 1 µm in size, with larger HSPs (>1 µm) being excluded, although these are discussed briefly wherever required. The terms “particles” and “HSPs” are interchangeably used in the manuscript.

## 2. Synthesis

Initial efforts to synthesize HSPs were focused on the use of micelles or reverse microemulsions, in which an amphiphilic polymer (e.g., cetyltrimethylammonium ammonium bromide; CTAB) is dissolved in water. The hydrophobic carbon chain repels the water molecules to minimize system energy via hydrophobic–hydrophobic interactions [27]. The hydrophilic ammonium (NH_4_^+^) head faces the bulk water phase. Silica is deposited by adding different types of silanes in the presence of a catalyst (by using a common sol–gel process based on the Stöber method) [28], resulting in the formation of a silica shell around the polymer micelles or emulsion droplets. The Stöber method, though commonly used for synthesis of solid silica particles, is also applied to deposit silica on different types of templates when the template is present in the solution. The Stöber method involves hydrolysis of silane molecules (e.g., tetraethyl orthosilicate (TEOS), sodium silicate, or tetramethyl orthosilicate (TMOS)) in an alcohol–water solution in the presence of a catalyst (e.g., ammonium hydroxide). The hydrolyzed molecules start polymerizing among themselves and make larger molecules (oligomers), which deposit on the template to make a silica shell. The polymer core is then removed by calcination, thus forming HSPs. In one example, reverse microemulsions of acidic water were formed in heptane in the presence of surfactant 1,2 bis (2-ethylhexyloxycarbonyl)-1-ethanesulfonate (AOT). This was followed by the addition of sodium silicate as a silica precursor, followed by calcination [29]. This resulted in the formation of HSPs that ranged from 1 to 10 µm. The HSP size was controlled by manipulating the concentration of the water phase and the reaction temperature, which in turn affected the microemulsion droplet size and the HSP size. Hah et al. [30] synthesized HSPs using phenyltrimethoxysilane (PTMS) alone without using any additional surfactant. Synthesis was performed in two steps. The first step was the formation of PTMS droplets in acidic aqueous conditions. Since phenyl groups are hydrophobic, they repel water. Therefore, PTMS creates emulsion droplets with phenyl on the inside and hydrolyzed methoxy groups on the outside. In the second step, ammonium hydroxide was added, resulting in condensation of hydrolyzed methoxy groups, leading to the formation of HSPs. Monodispersed HSPs were obtained with diameters ≈ 300–800 nm, internal cavities of 40–500 nm, and a ratio of hollow cavity/particle size ≈ 0.10–0.67. The HSP size and shell thickness were controlled via the reaction time and PTMS concentration. Since the process was surfactant-free, it could be scaled up, but this resulted in HSPs with thicker shells, so not much work was reported after the initial studies.

Small HSPs (100–200 nm) with shell thicknesses of 20–60 nm were synthesized by using water-in-oil reverse microemulsion, which was created using water and CTAB (surfactant) in dodecylamine (oil phase) [31]. Although small HSPs could be created, HSPs aggregation and low yield were the main issues. Similarly, surfactant-free water in oil (e.g., cyclohexane, dodecane, benzene, octane, and hexane) emulsions were used to make HSPs [32]. Several other organic solvents were exploited with and without surfactants [32,33,34].

Li et al. reported the surfactant-free synthesis of HSPs using ammonia solution and tetraethyl orthosilicate (TEOS) alone via the oil-in-water emulsion approach [35]. Initially, a small amount of TEOS was injected into an aqueous ammonia solution. This resulted in the TEOS forming into small droplets because of its insolubility in water. TEOS started polymerizing on the outer surface of droplets and more TEOS diffused from inside the droplets to complete the shells. Additional TEOS was added in a second step to complete the shell formation. The result was formation of HSPs ranging from 300 to 700 nm in size. However, the HSPs formed using this approach had incomplete shells and were polydisperse. HSPs ranging from 50 nm to 10 µm can be obtained by using a microemulsion or micelle approach; however, the associated low yield, high particle aggregation, and polydispersity have limited widespread use of this approach.

HSPs have also been synthesized by using organisms such as viruses and bacteria [36,37,38]. When using Gram-negative bacteria such as *E. coli*, water inside the bacteria acts as an initiator for hydrolysis of the silica precursor, TEOS. Additionally, the negative surface of the bacteria attracts positively charged ammonium ions, further aiding in deposition of negatively charged silicate molecules on the surface via the Stöber method, creating a silica shell. Viruses such as yeast and Gram-positive bacteria such as lactic acid bacteria were also used as templates to synthesize HSPs. Organism (bacteria or virus) templates can be of a variety of shapes, such as spherical, rod-shaped, curved rod-shaped, and spiral, and thus can provide HSPs of various shapes. However, organism-based templates have not attracted much attention because of their high cost and the less tunable sizes of the obtained HSPs (obtainable sizes: 1–5 µm), which are determined by the size of the organism template used.

Synthesis of HSPs using inorganic templates such as calcium carbonate [39,40,41], hydroxy apatite [42], and carbon particles [43] have also been explored. Silica deposition is performed using the common Stöber method, while the inorganic core is removed by either acidic dissolution [39,40,41,42] or by heating at high temperature [43]. This approach is effective, resulting in the formation of smaller (30–100 nm) HSPs; however, creating inorganic particles with good size control is a challenging and costly task. Furthermore, removal of inorganic particles is time consuming, and in most cases incomplete removal leaves behind some contamination of the inorganic templates.

Etching, a process of partially dissolving the substrate under acidic or basic conditions to create the desired shape of the same substrate, was also explored to create HSPs. Zhang et al. demonstrated HSP synthesis using preformed solid silica particles covered with polyvinylpyrrolidone (PVP) polymer [44]. Incubation of these PVP-covered silica particles in a basic (aqueous solution of NaBH_4_ or NaOH) solution resulted in etching of the silica core, while the outer silica shell was retained with PVP molecules attached. Similarly, other polymer or surfactants such as CTAB were also used to make HSPs using etching of solid silica particles [45]. Core etching occurs because of the lower stability of the pure silica core compared to the polymer-attached peripheral silica in alkaline conditions. Similarly, HSPs were synthesized by selective etching of solid silica particles with different organic functional groups in the core and peripheral region. In one example, initially formed cyano (–C≡N) or vinyl group (–CH=CH_2_)-terminated solid particles were coated with thiol-containing silanes. These hybrid solid particles were treated with basic solutions of NaOH or Na_2_CO_3_. The inner core (cyano- or vinyl-group-functionalized) was etched, while the thiol-functionalized outer peripheral part was stable to basic conditions, resulting in HSPs. Etching of the silica particle can provide size tunability. However, in several cases, etching has been an incomplete and time-consuming process.

Some unconventional techniques such as spray drying were also investigated [46,47]. In this work, preformed colloidal silica particles were organized into hollow microparticles by rapidly evaporating the droplets containing the colloidal silica particles. This approach looks promising because it is a continuous process instead of a batch process. However, the obtained HSPs were aggregated and polydisperse in size. Additionally, this technique gives colloidosome-like assemblies, in which small colloidal particles assemble at the interfaces of the emulsion droplets and bulk medium instead of resulting in true HSPs with a continuous shell made of amorphous silica.

Techniques such as ultrasonic spray pyrolysis were also explored [48,49]. These unconventional techniques can be used for large-scale and continuous HSP synthesis; however, control of the HSP size and shell thickness was poor. While most of the template-based synthesis approaches employ alkoxy silanes, successful use of low-cost precursor sodium silicate was demonstrated in this spray pyrolysis approach.

The most common strategy for synthesis of HSPs involves the use of polymer or copolymer particles (e.g., polystyrene, polyresorcinol, polymethylmethacrylate (PMMA), poly(methylmethacrylate-co-2-diethylaminomethyl methacrylate)) as templates [50,51,52,53,54,55]. This is followed by deposition of silica (using the Stöber method) using various silica precursors such as TEOS, TMOS, and sodium silicate. In the next step, polymer templates are removed by dissolving in organic solvents such as toluene or calcination at high temperature (≈550 °C). Because TMOS is highly reactive and prone to quick hydrolysis, it is less commonly used, whereas TEOS, being more stable to hydrolysis, is widely used. The polymer particle template-based approach is highly advantageous in providing HSPs in which the size is uniformly controlled, ranging from 50 nm to 1 µm, yet the yield of the synthesis process is quite low (1–3 cm^3^ HSPs are obtained per 100 mL of alcohol). More efforts should be made to increase the synthesis yield so that this approach can be more economically viable.

Not only have HSPs been created with a shell made of silica alone, but also hybrid HSPs with a shell made of two materials have been reported [56]. Wang et al. used an aerosol-based method in which a solution of ferric chloride, CTAB, sucrose, and a TEOS solution in ethanol were used. The ferric chloride–CTAB–sucrose was confined inside the rapidly forming silica shell when the aerosol droplets were passing through the heating furnace. During carbonization at 400 °C, vaporized carbon made from the sucrose formed the inner layer of the shell, whereas the silica formed the outer layer of the shell. Similarly, hybrid HSPs with composite shells made of silica and alumina were created by co-deposition of alumina and silica precursors on preformed polystyrene particles [57]. Recently, hybrid HSPs with a two-layered shell (inner layer silica and outer layer polymer or carbon) have been reported [58]. In this work, a polymer layer was deposited on preformed silica shell to a make silica–polymer two-layered shell, and by carbonizing the outer polymer layer, hybrid particles with a silica–carbon (two-layered) shell were obtained.

**Figure 1:**
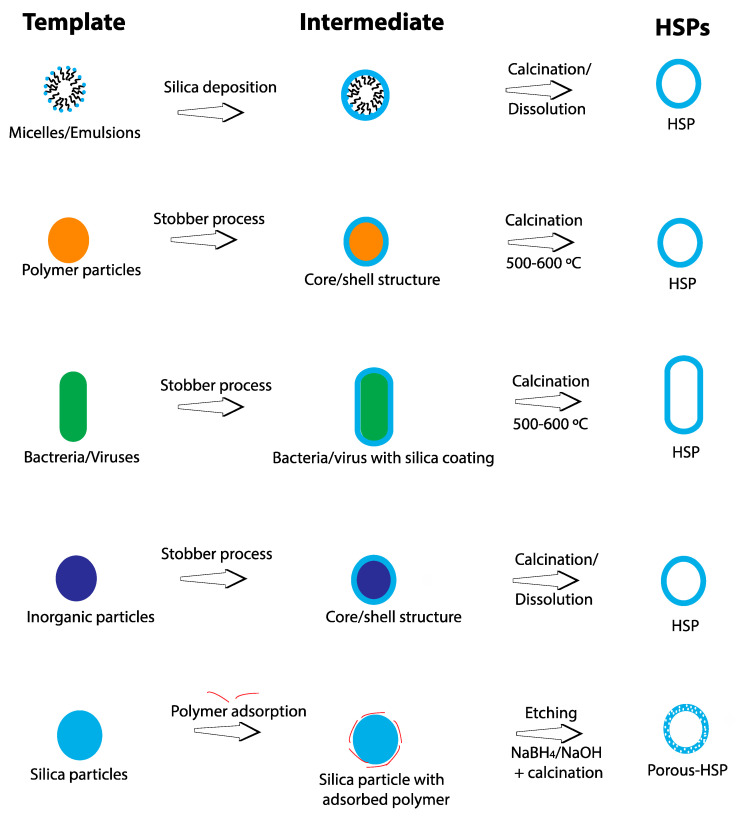
Schematic illustration of common strategies used for hollow silica particle (HSP) synthesis.

**Figure 2:**
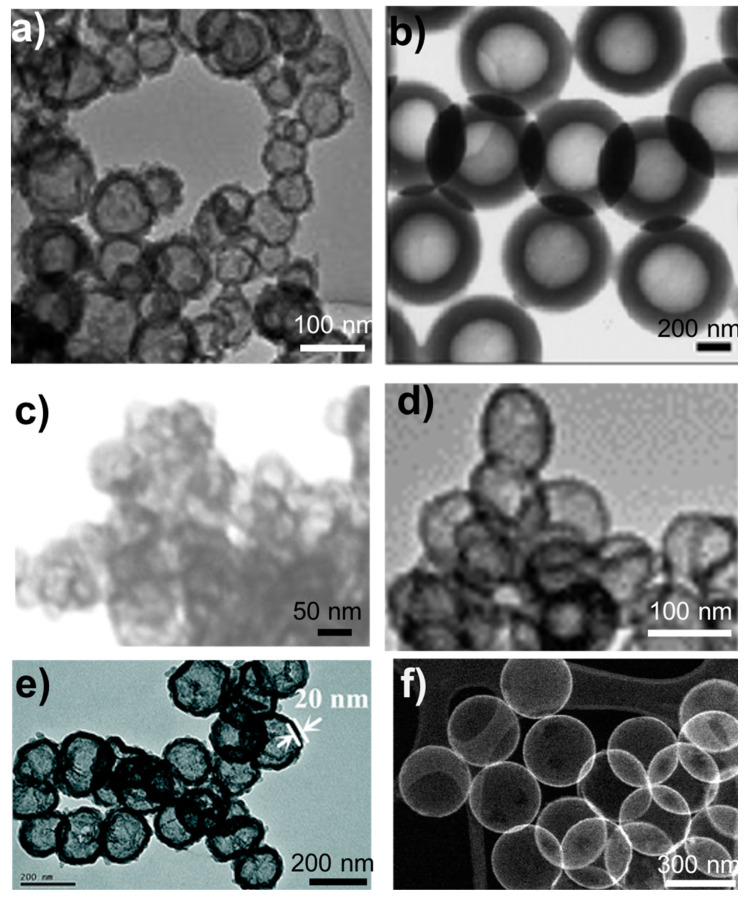
TEM images of representative HSPs synthesized by using (**a**) micelles templates [32], (**b**) surfactant-free emulsion [30], (**c**) calcium carbonate particle templates [41], (**d**) etching of solid silica particles [44], (**e**) carbon particle templates [43], and (**f**) polystyrene particle templates [58]. Reproduced from [30] with permission from Royal Society of Chemistry (2003), Reproduced from [43] with permission from Royal Society of Chemistry (2015), Reproduced from [58] with permission from Royal Society of Chemistry (2020). Reproduced from [32] with permission from Elsevier (2010). Reproduced from [41] with permission from Springer Nature (2018). Reproduced from [44] with permission from Elsevier (2012).

As mentioned in the introduction, any technique that involves removal of the template (e.g., polymer molecules, polymer particles, carbon or calcium carbonate particles) and etching (dissolution) of solid silica particles results in meso- or microporous particles. The removal of these materials either results in holes in the silica shell caused by gases escaping (as in the case of polymer particle templates) or removal of embedded polymer molecules, which leads to pore formation. Figure 1 depicts the common approaches to HSP synthesis, while Figure 2 shows TEM images of HSPs synthesized using representative approaches.

Several strategies can be applied to synthesize HSPs, each with its own advantages and disadvantages, as shown in Table 1.

## 3. Characterization

HSPs are characterized using common material science techniques. Scanning electron microscopy (SEM) imaging is the main technique employed to determine the HSP size, while transmission electron microscopy (TEM) helps in determining the shell thickness and shell type (solid or porous), in addition to the HSP size [13,23]. Brunauer–Emmett–Teller (BET) analysis is applied to measure the specific surface areas (surface area per unit of mass or volume) of mesopores present in the shells of the HSPs [35,41,59]. BET analysis utilizes the amount of gas (generally N_2_) adsorbed and desorbed at boiling point (77K) and different partial pressures in order to deduce the specific surface area of the porous material. BET analysis derives the specific surface area by calculating the amount of gas molecules required to make a monolayer from adsorption–desorption isotherms. TEM can also be employed for measuring the size of pores in the particle shell, however it only provides information about surface pores and not about the pores inside the shell. In order to measure the size and volume of the pores present in the mesoporous shell, Barrett–Joyner–Halenda (BJH) analysis is the technique of choice. Instead of focusing on monolayer adsorption of gas molecules, as is the case for BET, BJH analysis involves multilayer adsorption of gas molecules. When partial gas pressure is increased, gas molecules first fill (condense inside) the small pores. As the gas pressure is increased, all the pores get filled (or gas molecules condense in all pores). A gradual decease in pressure results in the reverse process––desorption of gas molecules from pores. From the adsorption–desorption isotherms generated during this process, the pore size and pore volume are calculated by applying BJH analysis. Knowledge of shell porosity is of utmost importance for applications such as drug delivery and catalysis, where HSPs are generally used either as containers or as high surface area supports. X-ray photoelectron spectroscopy (XPS) and Fourier transform infrared (FTIR) spectroscopy are applied to determine the types of functional groups (e.g., –NH_2_, –CH_3_, –C_2_H_5_, epoxide, and sulfide) on the HSP surface [21,51,60]. This information helps in the conjugation of HSPs with other materials (e.g., polymers, and drug delivery molecules) and in making hydrophilic or hydrophobic coatings. The thermal properties (e.g., thermal conductivity, thermal diffusivity, and heat capacity) of HSPs are measured by using either transient-state methods (e.g., transient plane source) or steady-state methods (e.g., heat flow meter) [61,62]. Nanoindentation and atomic force microscopy (AFM) are commonly employed to determine the mechanical properties (e.g., Young’s modulus and compressive strength) of particles [62]. Nanoindentation is an ensemble technique where a layer of particles is pressed, then based upon the area of the nanoindentor and the particle cross-section an average mechanical property (e.g., Young’s modulus of particles) is calculated, while AFM is a precise technique that can provide mechanical properties of individual particles. Knowing these mechanical properties is important for applications such as thermal insulation, as well as for battery electrodes or electrolytes. HSP surface features (e.g., roughness) are determined by using either AFM or TEM [23]. UV-Vis spectroscopy is applied to measure the optical properties (e.g., refractive index and visible transmittance) of HSPs [63,64,65,66]. Information about the optical properties of HSPs is highly useful in the fabrication of transparent coatings (e.g., antireflective coatings).

The minimum required characterization of particles depends on the desired application. For example, thermal insulation applications mainly require information about the particle size, shell thickness, and mechanical properties, while antireflective coatings need information about the particle size, refractive index, and visible transmittance. For drug delivery and catalysis applications, the minimum required information must cover the particle size and shell texture (porous or non-porous, pore size, and surface area). Similarly, energy storage applications require information about the mechanical properties and shell texture. Although the minimum required characterization information is different for different applications, in general the HSP size and shell thickness are the two main parameters used to characterize the HSPs in the literature.

Table 2 presents the general techniques used for characterization of different properties of HSPs.

## 4. Applications

### 4.1. Thermal Insulation

Thermal insulation materials are highly sought after for applications such as building envelopes, refrigerators, cryogenic storage of gases, and thermal energy storage systems [67,68,69,70]. Conventional thermal insulations materials include mineral wool, glass fiber, cellulose, polyurethane foams, and extruded polystyrene (PS) foams [71,72,73]. However, the thermal conductivities (0.026 to 0.040 W/m·K) of these materials are quite high, reducing their insulation capabilities. Silica aerogels are emerging insulation materials with a thermal conductivity range of ~0.014 to 0.020 W/m·K [74]. Unfortunately, aerogels are fragile, expensive, and lack scalability, hindering their widespread use. As an alternative, the use of HSPs in developing thermal insulation materials is an interesting research area [75]. The presence of hollow cavities inside HSPs results in very low thermal conductivities (0.02–0.03 W/m·K; depending on the particle size and shell thickness) [13,75,76,77]. When the hollow cavity is filled with air or any low conductivity gas, the overall heat transfer through HSPs becomes quite low as compared to solid silica or solid silica particles. The scientific principle behind this is that the overall solid conduction is minimal because of the very small amount of silica in the shell; materials such as air or gas inside the cavity contribute to lower thermal conductivity and improved insulation. Maximizing the benefit of this characteristic, several types of thermal insulation materials have been developed using HSPs as additives. For example, thermally insulating thin films or coatings have been developed [78,79,80,81,82] by adding HSPs to polymers, such as polyurethane or polyisocyanurate. These films or coatings generally have thermal conductivity that is 50–70% lower than the original polymers used as the matrix. The size of the added HSPs depends on the intended application of the composite film. For example, transparent films need small (<100 nm) particles, while large particles (>100 nm) can be used in opaque films. This field is growing fast, as these types of films have extensive applications in windows in buildings and vehicles.

HSPs can also be used to make aerogel-like materials. In these materials, the cavity is preformed, so less effort is required to avoid shrinkage of pore walls, which is the main challenge in conventional aerogels [83]. Aerogels made of HSPs could be a new class of thermal insulation material. HSPs have also been tested as additives in epoxy and silicone to improve their thermal insulation properties [62]. Although not reported before, we envisage that HSPs can be used to make hybrid insulation materials by mixing with other solid inorganic particles (e.g., silica, titania, carbon, etc.). The thermal conductivity of HSPs generally depends upon four parameters: (1) shell thickness, (2) cavity size, (3) shell quality (completely or incompletely formed), and (4) particle purity (with or without any debris of silica grains or solid silica particles in the sample). Thicker shells result in more solid silica in the system or higher solid thermal conduction. This increases the overall thermal conductivity of the system, thus lowering the thermal insulation value of the particles. Therefore, a thin shell is preferred for thermal insulation applications. The particle cavity size is an important factor in controlling thermal properties. The smaller the cavity size, the lower the thermal conductivity. The small size of the cavity leads to minimal heat transfer through the air trapped inside the cavities. This is because the air molecules have more chances of colliding with the cavity walls than colliding with each other. This is known as the Knudsen effect, in which transferred energy is lower in collisions between air molecules and walls than in collisions between air molecules [84].

A small cavity size means a higher number of HSPs required for the same volume of air, which indirectly increases the amount of solid silica in the system, thus increasing the overall thermal conductivity. Therefore, an optimized cavity size is required to obtain the maximum achievable thermal insulation from HSPs. Additionally, the cavity size will be different if the standalone thermal insulation material used for HSPs is made when compared to the use of HSPs as an additive in polymers to lower their thermal conductivity. The main cause of this difference is that in the standalone material, air trapped inside the interstitial space between the particles also plays a role in lowering the thermal conductivity, but when used as additive, this interstitial space is missing and only the cavity inside the particles plays a role in reducing the thermal conductivity.

In addition to the cavity size and shell thickness, the particles’ surface features can also affect their thermal insulation properties. For example, a rough surface can result in more heat phonon scattering when compared to smooth surfaces, which is good for thermal insulation applications [76].

HSP purity, the final factor discussed here, is the most important. If the sample has broken HSPs or some solid silica particles formed as coproducts, then the overall performance of the HSPs will be deteriorated.

**Figure 3:**
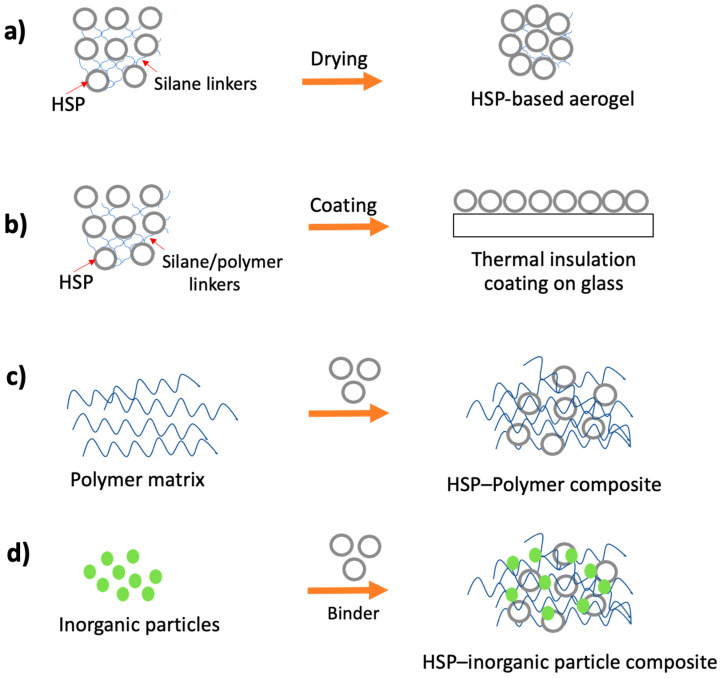
Schematic illustration of common existing and possible HSP-based thermal insulation materials. (**a**) an aerogel made by crosslinking HSPs with silane linkers, (**b**) a coating made by incorporating HSPs in polymer matrices, (**c**) thermal insulation material based on polymer–HSPs composite, (**d**) A hybrid insulation material made by mixing HSPs with other inorganic particles.

HSPs, being inherently stable at high temperatures (silica melting point ≈ 1710 °C), are good candidates for high temperature thermal insulation applications (e.g., insulation of concentrated solar power storage tanks and pipes [85], high-temperature fuel cells [86], where conventional insulation materials based on foams or cellulose fibers are not suitable. Polymer and carbon hollow particles can also be used for thermal insulation applications [11,14]. Although polymer hollow particles can provide better insulation (lower thermal conductivity) compared to HSPs, polymer hollow particles have low mechanical properties (generally crumble under their own weight) and are flammable. Similarly, carbon hollow particles have good mechanical properties (although less than HSPs) and are stable at higher temperatures (≈250 °C; carbon oxidation temperature ≈ 450–500 °C) [87]; however, hollow carbon particles have higher thermal conductivity and are not suitable for thermal insulation applications [58]. Figure 3 illustrates four common and possible methods for making thermal insulation materials using HSPs.

### 4.2. Drug Delivery

**Figure 4:**
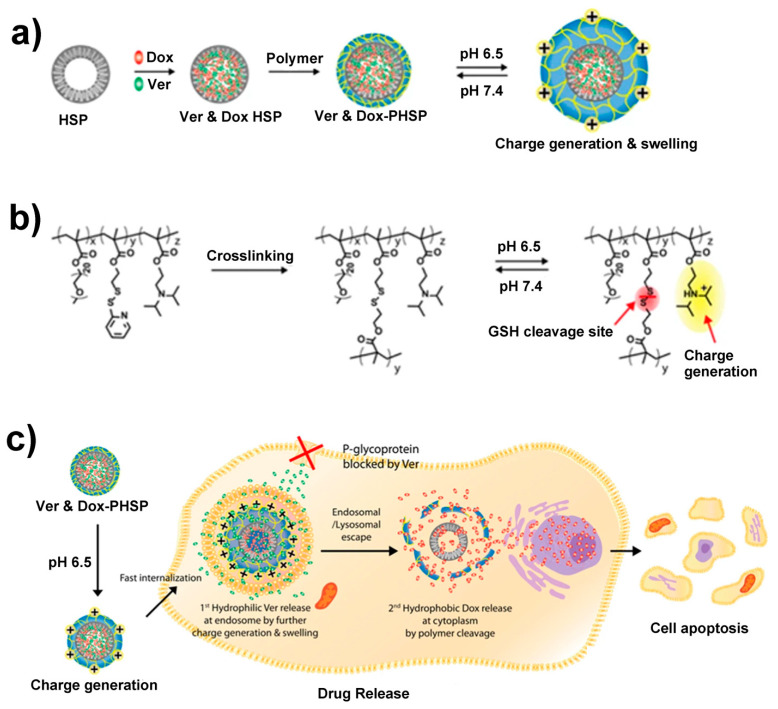
(**a**) Scheme for preparation of drug-loaded PEG(polyethylene glycol)–PDS(pyridine disulfide)–DPA{2-(diisopropylamino) ethyl methacrylate} copolymer-coated HSP (PHSP), (**b**) disulfide crosslinking, and pH-dependent cationic charge reversal by the protonation of the diisopropylamino group. (**c**) Schematic illustration of the cellular uptake, endosomal escape, and glutathione (GSH)-mediated drug release. Under tumoral acidic conditions (pH 6.5), positive charge reversal results in a fast cellular uptake. Further increase of the positive charge of the polymer in the endosome (pH 5.0–5.5) induces the swelling of the polymer, followed by the release of hydrophilic Ver, facilitating the endosomal escape of the nanoparticles. In the presence of intracellular GSH, the second drug, hydrophobic Dox, is released. Adapted from [59] with permission from Springer Nature (2017).

For drug delivery to a specific organ (or tissue), the main drug delivery carriers include, micelles, liposomes, and polymeric particles. Although inexpensive, these carriers suffer from poor chemical stability and short circulation time in the body, and thus in several cases get cleared from the body or destroyed even before delivering the drug [88]. Silica particles (HSPs), being highly chemically stable and with longer circulation lifetimes in the body, could be alternative drug delivery carriers [88]. The unique properties—including their tunable cavity size, low density, large surface area, micro or mesoporous shell, easy-to-functionalize surface, and less or no toxicity compared to other nanomaterials such as carbon nanotubes or fullerenes, make HSPs ideal carriers for drug delivery. All of these properties are difficult to find together in any other material, which further enhances the importance of HSPs. The hollow cavity provides more space for loading the drug molecules, while the mesoporous shell allows the diffusion of the drug through the shell. The large surface area is suitable for loading the drug molecules on the surfaces by physical or chemical adsorption to the inner and outer surfaces of HSPs and to the inner pore walls. The silica shell surface inherently has –OH groups that can be easily functionalized with other molecules or drugs, making this a preferred material. Most importantly, the key prerequisite for any material used as a drug delivery vehicle is that it is not toxic to the recipient and that it degrades over time after delivering the drug. Amorphous silica easily degrades in water over time [89] and silica particles are easily removed from body through the kidneys [88]. HSPs have been demonstrated as carriers for the delivery of drugs such as doxorubicin hydrochloride, cytochrome C, polymyxin B, camptothecin, and cytosine-phosphodiester-guanine oligodeoxynucleotide [90,91,92,93,94,95]. Besides their use in conventional drugs, HSPs have also been shown to be useful as carriers for unconventional vaccines, such as virus-like particles (VLPs). These particles contain one or more viral structural protein, but they lack viral genetic material. VLPs for foot and mouth disease in animals were adsorbed on HSPs and injected into guinea pigs. VLPs adsorbed in HSPs showed better immune response than those with the VLP–Freud’s complete adjuvant. These studies open more pathways for additional investigation of HSPs as vaccine-loading carriers [96]. In addition to simple loading of drugs without requiring any sophisticated chemistry, some efforts have been made to achieve the controllable release of drugs from particle cavities. For example, Palanikumar et al. demonstrated a pH-triggerable and redox switchable release of drugs from HSPs [59]. Two drugs, doxorubicin (Dox) and verapamil-HCl, were entrapped inside the cavity and were also attached to the pores inside the HSP shell (100 nm diameter and 15 nm shell thickness) by physical interaction. Positively charged self-crosslinkable random copolymer containing pyridine disulfide (PDS), 2-(diisopropylamino) ethyl methacrylate (DPA), and polyethylene glycol (PEG) were used to coat the negatively charged, drug-loaded HSPs. The polymer was crosslinked via sulfide–sulfide bond formation. Once the particle reached the acidic cancer cells, the diisopropylamino groups of crosslinked polymers became positively charged and swelled, resulting in the outer coating opening and releasing the drugs. Additionally, glutathione molecules present inside the cells resulted in cleavage of the disulfide bonds, which loosened the crosslinked polymer network inside the cancer cells. Figure 4 demonstrates the controlled release of hydrophobic (doxorubicin) and hydrophilic (verapamil-HCl) drugs by using crosslinked, polymer-coated HSPs (PHSPs). Additionally, HSPs filled with perfluoropentane gas were also employed as ultrasound contrast agents, whereby perfluorpentane generates the ultrasound signal and HSPs act as the gas carrier. Compared to the conventional liposomal or polymeric gas carriers, the HSPs showed longer in vivo stability and a better imaging lifetime [56,97,98,99].

### 4.3. Energy Storage

**Figure 5:**
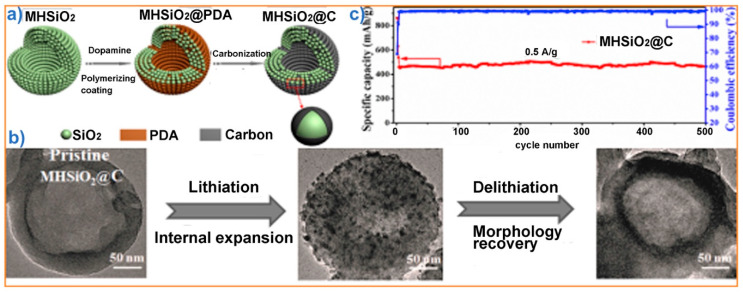
Example showing the use of HSPs made by assembling carbon-coated nanoparticles as battery anodes: (**a**) formation of carbon-coated HSPs; (**b**) dilithiation cycle showing expansion and contraction of carbon-coated HSPs; and (**c**) plot showing the cyclic stability of the anode. PDA: polydopamine. Adapted from [101], with permission from Elsevier (2017).

New applications of HSPs as electrolyte stabilizers or anodes in lithium ion batteries have been reported [17,99,100,101,102]. In one such example, Zhang et al. used HSP-based films filled with liquid electrolytes as solid electrolytes that show lithium-ion conductivities in the order of approximately 1 mS, while being mechanically stable and mitigating the penetration of dendrites [99]. Cao et al. demonstrated that HSPs embedded in porous carbon are promising anodes for lithium ion batteries. Such electrodes can provide a specific capacity of 910 mA h g^−1^ at a rate of 200 mA g^−1^ after 150 cycles, while exhibiting reasonable rate capability [100]. HSPs made of carbon-coated silica nanoparticles and several other combinations of carbon and HSPs have also been demonstrated as battery anodes (as shown in Figure 5) [99,100,101,102]. These initial results were encouraging, but at present the use of HSPs in battery applications is not attracting much interest because of the low ionic and electrical conductivity of HSPs. More research is required to investigate the possible use of HSPs in batteries as scaffolds for solid electrolytes or as composites with carbon or other electrode materials.

Besides their use as anode materials for batteries, HSPs have also been explored as additives in the polymer matrix to make electrolytes with better mechanical and electrochemical properties and with good thermal conductivity. In one example, sulfonated HSPs filled with sulfuric acid were added to a polyvinyl alcohol (PVA) matrix. The symmetrical supercapacitor assembled using particle–PVA electrolytes showed a specific capacitance value of 147 F g^−1^ at 0.5 A g^−1^ [103]. HSPs have also been explored as encapsulation containers for phase change materials (PCMs), in which the particles are filled with the PCM, then the shell keeps the PCM confined and does not allow its free flow when it melts [104,105,106]. PCMs are materials that release or absorb large amounts of energy when changing phases. For example, paraffin absorbs energy and converts it into the liquid phase, which converts back to the solid phase with the release of energy. PCMs are commonly incorporated in building materials to lower the heating or cooling costs of buildings [104,105,106]. HSP-based encapsulation of PCMs is a promising method, but further improvements are required to increase the thermal conductivity of the silica-based shell. These improvements will help to achieve a high charge–discharge rate for PCMs. Furthermore, loading HSPs with PCM materials is a challenging task, so more techniques are required to enhance the loading capacity of PCMs. One possible way to increase the thermal conductivity of the HSP shell may include making a hybrid shell with embedded carbon or another high conductivity material.

### 4.4. Functional Coatings

HSPs have been used in several types of transparent and opaque functional coatings, such as antireflective, superhydrophobic, and superhydrophilic coatings. The low refractive index (1.28) of HSPs compared to solid silica particles (1.46) makes them ideal candidates for transparent functional coatings, especially on glass or polymer surfaces [63]. To achieve an antireflective coating, the refractive index of the coating must be lower than the substrate on which the coating is applied. A lower refractive index helps to achieve a refractive index gradient from air (1.0029) < coating (1.2–1.3) < glass (1.5–1.65). This helps to avoid a sudden refractive index change, thus minimizing the reflection [64,65,66]. Better gradients result in better antireflective properties. A large volume of air entrapped inside the HSPs lowers their refractive index compared to solid silica particles, glass, or polymer surfaces. This results in a smooth transition of the refractive index from air to coating to substrate. By modifying the particle size and shell thickness, coatings with a range of antireflective properties have been explored.

**Figure 6:**
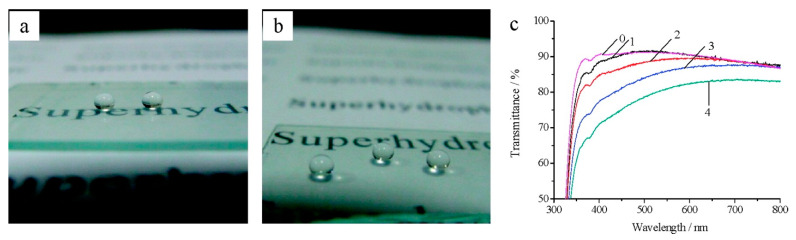
Digital photos of glass slides with superhydrophobic coatings prepared by dip-coating the APTS-modified HSPs: (**a**) APTS 0.70 wt % and (**b**) APTS 0.90 wt %. (**c**) Transmission spectra of coatings on glass slides: (0) bare glass slide and (1−4) slides prepared by dip-coating the 0.30 wt %, 0.50 wt %, 0.70 wt %, and 0.90 wt % APTS sols. Adapted with permission from [107]. Copyright (2012) American Chemical Society.

Antireflective coatings are generally made by mixing the HSPs with polymer or hydrolyzed silane slurries to firmly bind the coating with the substrates (polymer or glass). The same antireflective coatings can be made as superhydrophobic coatings by applying fluorosilanes on the surfaces of HSP-based coatings, by mixing the fluorosilanes during the coating formation into the slurry, or by modifying the HSPs with fluorosilanes [64]. If no fluorosilanes are applied, then the same HSP-based coating become superhydrophilic. These superhydrophobic and superhydrophilic coatings are used in applications such as self-cleaning surfaces. Similarly, antireflective coatings are highly desired in applications such as antiglare glasses, TVs, and camera screens. Figure 6 shows a transparent superhydrophobic coating made by dip-coating 3-aminopropytriethoxysilane (APTS)-modified HSPs on glass slides, followed by chemical vapor deposition of fluorosilane on these dip-coated HSPs [107]. The coating had a water contact angle of 146° and visible transmittance of 83.7%. The water contact angle is a measure of the surface wettability with water. If the water contact angle is ≤90° the surface is hydrophilic, and if it is ≥90° the surface is hydrophobic. The higher the contact angle, the higher the hydrophobicity of the surface.

### 4.5. Catalysis

Because silica is chemically quite stable, it has been used extensively as a support for catalysts. Other characteristics that make silica a suitable candidate as a support material are its easy-to-functionalize surface, low cost, high surface area, and high thermal stability (melting point ≈ 1710 °C). HSPs have been used as catalytic supports for several catalysts, such as Ag, Pt, enzymes, MnO_2_, TiO_2_, and Ni [108]. There are two main approaches for incorporating catalysts into HSPs. In the first approach, catalysts are impregnated inside the preformed HSPs [109,110,111]. However, in most cases, the catalyst attaches to the outer surface of the shell and does not enter the cavity, shell pores get clogged, and several repetitions are required to impregnate a sufficient amount of catalyst inside the cavity. In the second approach, catalyst nanoparticles are preformed, followed by deposition of the silica shell [111,112,113]. While this approach is better than the first, it still requires multiple steps; therefore, further research is needed to decrease the number of steps.

TiO_2_ particles are known for their photocatalytic decomposition of organic materials, which makes them suitable candidates for self-cleaning TV screens or windows or as a standalone photocatalysts for cleaning indoor air. Ikeda et al. encapsulated TiO_2_ nanoparticles inside HSPs [114]. TiO_2_ nanoparticles were coated with a carbon layer, followed by a silica shell. The carbon layer was removed by heating, resulting in unbound TiO_2_ nanoparticles within the mesoporous hollow particle cavity, creating a TiO_2_–void–silica configuration. Organic molecules or substrates can diffuse through the mesoporous shell. This design allows accessibility to maximum active sites on the TiO_2_ nanoparticles without compromising the number of available sites, which occurs when there is no void and the silica shell is in direct contact with the TiO_2_ particles. The photocatalytic activity was analyzed by comparing the gas-phase decomposition of acetaldehyde and the liquid-phase decomposition of acetic acid and poly(vinyl alcohol). It was observed that by using such encapsulated TiO_2_ nanoparticles, the photo catalytic activity remained similar to that of unencapsulated pristine catalytic particles. Additionally, since the shells were micro- or mesoporous, only small substrates were allowed to reach the TiO_2_ catalytic sites, thus making these encapsulated particles suitable for size-selective photocatalysis.

**Figure 7:**
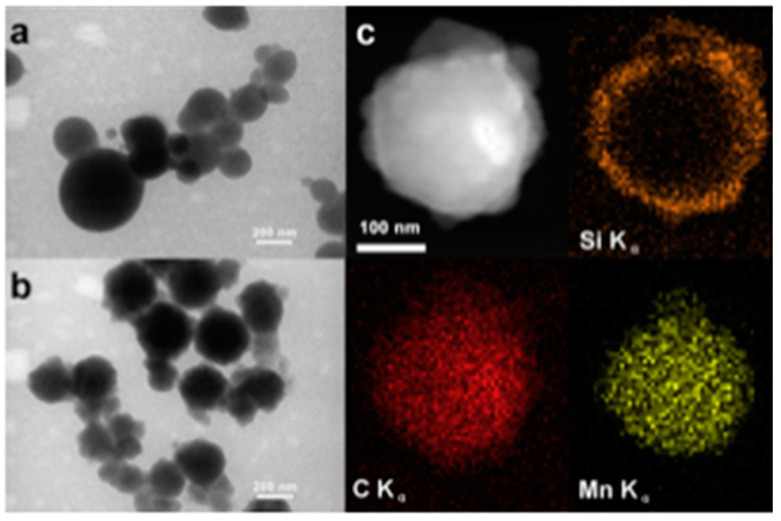
**(a**) TEM image of PAA-Mn aggregate particles before silica coating, (**b**) TEM image of PAA-Mn aggregate particles after silica coating, (**c**) STEM image and Si, C, and Mn EDX mapping images of a single particle after silica coating. Reproduced from [116] with permission from Elsevier (2013).

HSPs with silver chloride incorporated inside their mesoporous shells were used to make antibacterial polymer–HSPs composite coatings [115]. The antibacterial activities of the coatings were tested against candida albicans (ATCC 10231) and streptococcus mutants (ATCC 25175). These coatings showed improved antibacterial activities, high hardness, and acceptable adhesion to the substrate. In another approach, manganese oxide (MO) nanoparticles were encapsulated inside the mesoporous HSPs [116]. Initially, the MO nanoparticles were mixed with polyacrylic acid (PAA) to form MO-PAA aggregates. Silica shell was deposited on the MO-PAA aggregates using the Stöber process. Calcination at >400 °C resulted in the removal of the PAA contents, leading to loosely bound MO nanoparticles inside the mesoporous silica shell (Figure 7). MO particles encapsulated in the mesoporous hollow silica shells were observed to have more catalytic efficiency than pristine solid MO particles. Although tremendous progress has been made using HSPs as catalyst supports, the higher manufacturing cost of small HSPs (<1 µm) is still an unresolved challenge.

## 5. Outlook and Future Perspectives

There are several synthesis strategies for HSPs, however most require the initial formation of a template (micelle, emulsion, particle etc.), followed by deposition of silica via the Stöber process removal of the template via heating or dissolution. These processes are tedious and time-consuming, and in almost all cases the reaction yields are very low. Some template-less strategies involving TEOS alone have been devised, but the less control of the particle size and the low yield have hindered their widespread use. To make HSPs industrial-scale entities, a low-cost, high-yield manufacturing process is needed. Continuous manufacturing processes (not batch processes) are envisioned to be the future of HSP synthesis. Some of these processes have been tried, such as bubble-based, spray drying, and spray pyrolysis processes [46,47,48,49,117], but these processes still need more research to increase the control of the particle size and to ensure the use of inexpensive chemicals. The main focus has been on the synthesis of HSPs with shells made of silica alone, but new research areas are focusing on hybrid shells made of two materials. HSPs with hybrid shells (e.g., an inner silica layer and outer biocompatible polymer layer) can provide new useful properties, such as improved drug delivery and ultrasound contrast agents. Similarly, hybrid HSPs containing outer polymer layers could act as next-generation thermal insulation [58].

The use of HSPs has been demonstrated in several applications, especially drug delivery and catalysis. However, most of these applications are still at the lab scale and have not yet gone beyond publication of results. Therefore, more research is required to confirm if the use of HSPs in these applications is economically viable and advantageous compared to the incumbent technologies. The main focus is on the use of HSPs in drug delivery, however, new research areas such as thermal insulation and energy storage are gaining more attention. In thermal insulation, so far only conventional strategies involving the addition of HSPs to polymer matrices for thermally insulating composites have been investigated. There are several other possible thermal insulation applications that still need to be explored, such as HSP-based insulation for high-temperature thermal energy storage tanks. Loose HSPs can be a respiratory health hazard, so most of applications will include the addition of HSPs to other materials or crosslinking them with different binders. Therefore, powder HSPs as standalone thermal insulation products do not seem plausible at this stage. Similarly, for energy storage applications, more research will be needed to make HSPs ionically and electrically more conductive.

## Figures and Tables

**Table 1:** Advantages and disadvantages of HSP synthesis strategies.

No.	Strategy	Advantages	Disadvantages	References
1.	*Polymer micelles/emulsions*	Good for synthesis of small HSPs Well-established synthesis process	Less control of particle size Low yield	[27,28,29,30,31]
	Surfactant examples ^*^: CTAB, PVP, and PTMS			
2.	*Inorganic particles as templates*	HSPs size control	Incomplete dissolution of inorganic core Time-consuming High cost Low yield	[39,40,41,42]
	Examples: carbon, calcium carbonate, and hydroxy apatite			
3.	*Polymer particles as templates*	HSP size control Well-established process	Low yield High cost Solvent wastage	[50,51,52,53,54,55]
	Examples: polystyrene and polyresorcinol			
4.	*Solid silica particle etching*	HSP size control Well-known chemistries	Less control of hollow cavity size Time-consuming	[44,45]
5.	*Spray drying*	Potential for scale up	Less HSP size control Needs preformed silica nanoparticles	[46,47]
6.	*Spray pyrolysis*	Potential for scale up	Polydisperse HSPs	[48,49]
7.	*Bacteria/virus templates*	A wide range of available template shapes	Costly Less scalable Less HSP size control	[36,37,38]

* CTAB: Cetyltrimethylammonium ammonium bromide; PVP: Polyvinylpyrrolidone; PTMS: Phenyltrimethoxysilane

**Table 2:** Techniques and specific properties characterized.

No.	Technique	Property Characterized	Application	References
1.	SEM ^*^	Size and surface features (e.g., roughness)	Composites	[13]
2.	TEM ^*^	Size, shell thickness, shell texture (e.g., solid or porous)	Composites	[13,23]
3.	BET ^*^ and BJH ^*^ analysis	Shell pore size, pore volume, and surface area	Drug delivery and composites	[35,41,59]
4.	XPS ^*^, FTIR ^*^	Surface chemistry	Composites, drug delivery, thermal insulation, battery anodes	[21,51,60]
5.	Thermal conductivity measurement techniques, such as transient plane source	Thermal properties (e.g., thermal conductivity, thermal diffusivity, and heat capacity)	Thermal insulation materials (composites)	[61,62]
6.	Nanoindentation	Mechanical properties (e.g., Young’s modulus, compressive strength)	Composites	[62]
7.	AFM ^*^	Size, surface characteristics, and mechanical properties (Young’s modulus)	Composites and functional coatings	[23]
8.	UV-Vis ^*^	Optical properties (e.g., reflectivity, visible transmittance, and opaqueness)	Functional coatings, e.g., reflective or antireflective coatings, superhydrophobic coatings	[63,64,65,66]

^*^ SEM: Scanning electron microscope; TEM: Transmission electron microscope; BET: Brunauer–Emmett–Teller analysis; BJH: Barrett–Joyner–Halenda analysis; XPS: X-Ray photoelectron spectroscopy; FTIR: Fourier transform infrared spectroscopy; AFM: Atomic force microscope; UV-Vis: Ultraviolet-visible light.
